# Elevated 12,13-diHOME level in maternal and umbilical cord blood complicated with preeclampsia

**DOI:** 10.3389/fendo.2024.1445475

**Published:** 2024-10-08

**Authors:** Tomohiro Yoshida, Kohei Kitada, Kensaku Nakai, Ryo Uemura, Yasushi Kurihara, Mie Tahara, Akihiro Hamuro, Akemi Nakano, Takuya Misugi, Daisuke Tachibana

**Affiliations:** ^1^ Department of Obstetrics and Gynecology, Osaka Metropolitan University Graduate School of Medicine, Osaka, Japan; ^2^ Department of Obstetrics and Gynecology, Izumiotsu Municipal Hospital, Osaka, Japan; ^3^ Department of Obstetrics and Gynecology, Osaka City General Hospital, Osaka, Japan

**Keywords:** pregnancy, lipid metabolism, preeclampsia, fetal growth restriction, 12,13-diHOME

## Abstract

**Background:**

Preeclampsia (PE) is a condition in pregnancy characterized by hypertension and proteinuria, thus leading to severe complications for both mother and fetus, including fetal growth restriction (FGR). However, there are still unclear aspects regarding the pathogenesis, prevention, and treatments. This study aimed to elucidate the characteristics of lipid metabolism in maternal and umbilical cord plasma complicated with PE using liquid chromatography-mass spectrometry (LC-MS).

**Method:**

The study included singleton pregnant women at Osaka Metropolitan University Hospital from March 2023 to February 2024. PE was diagnosed based on new-onset hypertension after 20 weeks of gestation and other symptoms such as proteinuria and organ dysfunction. FGR was defined by ultrasound measurements below -1.5 standard deviation (SD). Plasma samples were collected from maternal and umbilical cord blood within 24 hours before delivery. Lipid metabolites were comprehensively analyzed using LC-MS, and the lipokine 12,13-diHOME, identified as elevated in the comprehensive analysis, was quantified. Immunohistochemistry was conducted on placental samples to assess soluble epoxide hydrolase (sEH) expression.

**Results:**

The study involved 31 participants, with 20 in the control group and 11 in the PE group. A comprehensive analysis of maternal plasma samples identified a significant increase in 12,13-diHOME levels in the PE group compared to the control group. Quantification of 12,13-diHOME showed a significant increase in maternal plasma, umbilical venous plasma, and umbilical arterial plasma in the PE group compared to the control group (*p* = 0.007, *p* = 0.008, *p* = 0.005). PE with FGR showed significantly higher 12,13-diHOME concentrations in the umbilical arterial/venous ratio compared to the PE without FGR group (*p* = 0.03). Negative correlations were observed between 12,13-diHOME levels and birth weight in the PE group. Immunohistochemistry did not show significant differences in the sEH expression between the groups.

**Conclusion:**

This study demonstrated that 12,13-diHOME levels were significantly elevated in maternal and umbilical cord blood in PE patients, particularly in PE with FGR. Elevated 12,13-diHOME may reflect the progression of placental ischemia due to PE pathogenesis. This lipid metabolite could serve as a marker for the severity of preeclampsia, thus providing new insights into perinatal lipidomics and the potential role of 12,13-diHOME in PE.

## 1 Introduction

Preeclampsia (PE) is characterized by hypertension, proteinuria, and organ failures associated with maternal vascular damage. It affects 2-8% of pregnant women and often worsens as long as the pregnancy continues (1, 2). In cases of severe PE who present with further high blood pressure (systolic ≥ 160 mm Hg or diastolic of ≥ 100 mm Hg), the patient will develop fatal conditions such as liver dysfunction, renal insufficiency, thrombocytopenia, pulmonary edema, cerebral or visual disturbance, or eclampsia (3). Furthermore, maternal vascular damages and circulatory pathophysiology may frequently lead to fetal growth restriction (FGR) (4). Conservative treatment may be an option until the fetus can survive outside the uterus, but if termination of the pregnancy becomes necessary, there is a risk of the fetus being premature (5). Thus, PE is highly associated with maternal and/or fetal morbidity and mortality (6).

The pathogenesis of PE is thought to be caused by inadequate placental perfusion (7). Shallow invasion of cytotrophoblast into the uterine spiral artery results in placental ischemia and malperfusion owing to the inadequate remodeling of the spiral artery (7). This condition is exacerbated by inflammation, immune responses, and oxidative stress, thus contributing to maternal systemic vasoconstriction and further feto-placental malperfusion. It may also trigger life-threatening events for mothers and fetuses (8, 9).

Various factors have been shown to be involved in the pathogenesis and pathophysiology of PE (10). Recent articles are adding novel understanding of the roles of several lipids and lipid metabolites in the vascular atherosclerosis and damage modifications (11, 12). The physiological lipid increase in maternal plasma is well known and it is conceptually thought to be caused by increased fetal demand, although the detailed mechanisms are not fully understood. The purpose of this study is to analyze changes in lipid metabolism in patients with PE in detail using liquid chromatography-mass spectrometry (LC-MS), to identify lipid profiles associated with PE, and to clarify their pathophysiological significance. Through this, the study aims to provide a foundation for the discovery of new biomarkers for the diagnosis, prevention, and treatment of PE, as well as to deepen the understanding of the disease’s pathology.

## 2 Materials and methods

### 2.1 Study participants and diagnosis of PE, and FGR

The study targeted singleton pregnant women who gave informed consent for research and delivered at Osaka Metropolitan University Hospital from March 2023 to February 2024.

PE was defined as follows: new-onset hypertension after 20 weeks of gestation (≥ 140/90 mmHg) and symptoms of proteinuria (≥ 300 mg/day or urine protein/creatinine ratio ≥ 0.3), renal insufficiency (creatinine > 1 mg/dL), liver disease without underlying conditions (aspartate aminotransferase (AST) > 40 IU/L or alanine aminotransferase (ALT) > 40 IU/L), neurological complications (eclampsia, cerebral hemorrhage, stroke, severe headache), hematological complications (platelet count < 150,000/dL, disseminated intravascular coagulation, hemolysis), or uteroplacental insufficiency (FGR, abnormal umbilical artery doppler waveform analysis) (13). FGR was defined as standard deviations (SD) of an estimated fetal body weight below -1.5, based on Japanese standards for ultrasound measurement of Japanese fetuses (14).

### 2.2 Sample collection

Plasma samples were obtained from the maternal vein within 24 hours before delivery and umbilical cord blood samples were obtained from the umbilical artery and umbilical vein at birth. These samples were then centrifuged at 15,000 rpm for 10 minutes at 4°C to separate the plasma, which was then stored at -80°C until analysis.

### 2.3 Comprehensive analysis by LC-MS

Comprehensive analysis was conducted using maternal blood samples. We investigated 196 Polyunsaturated Fatty Acids (PUFAs) metabolites using the Liquid Chromatography Tandem Mass Spectrometry (LC-MS/MS) Method Package for Lipid Mediators Ver.3 (Shimadzu, Kyoto, Japan) (15).

Plasma samples (30 µl) were diluted 300 µl with 0.1% formic acid in methanol containing an internal standard mixture (20 ng/ml of prostaglandin E2 [PGE2]-d4, 20 ng/ml of leukocyte triene B4 [LTB4]-d4, and 200 ng/ml of arachidonic acid [AA]-d8) and shaken for 5 minutes.

The supernatant was then loaded onto a solid-phase extraction cartridge (Strata-X, Phenomenex, Torrance, CA, USA) and washed sequentially with 1 ml of 0.1% formic acid in water, 15% ethanol in 0.1% formic acid/water, and hexane. The cartridge was eluted with 300 µl of methanol containing 0.1% formic acid using a positive pressure manifold such as the Bitoge^®^ PRESSURE +48 (Biotage, Uppsala, Sweden), and the solvent was evaporated using an automated solvent evaporation system such as the TurboVap^®^ LV (Biotage, Uppsala, Sweden). The residue was then dissolved again in 30 µl of methanol. Finally, 5 µl of the dissolved sample was analyzed on an LC-MS system consisting of a NexeraTMX2 system and a triple quadruple mass spectrometer LCMS-8060 (Shimadzu, Kyoto, Japan) (15). Chromatographic separation was achieved on a reversed-phase column (ACQUITY UPLC BEH C18, 1.7 µm, 2.1×100 mm, Waters Corporation, Milford, MA) at a flow rate of 0.6 ml/min. For mobile phases A and B, 0.1% formic acid and acetonitrile in water were used, respectively. Sample cooler and column oven temperatures were set at 5°C and 40°C, respectively. Peak analysis was performed using LabSolutions software (Shimadzu, Kyoto, Japan). The limit of detection was set at a signal-to-noise ratio of less than 3. Values below this limit were treated as “0” and included in the analysis (16).

### 2.4 Quantification method

Quantification was performed using the same comprehensive analysis method. A calibration curve was constructed, and quantification was conducted using a known concentration of 12,13-dihydroxy-9Z-octadecenoic acid (12,13-diHOME) solution. In addition to the three previously used internal standard substances (PGE2-d4, LTB4-d4 and AA-d8), 12,13-diHOME-d4 (Cayman Chemical Co. Ann Arbor, MI, USA) was employed for more precise quantification (17). Following the same comprehensive analysis method as described previously (15), 12,13-diHOME-d4 was added as an internal standard and at a concentration of 20 ng/ml. Peak analysis was conducted using LabSolutions software and the obtained calibration curve was used to quantify the concentration of 12,13-diHOME.

### 2.5 Immunohistochemistry

After conducting a pathological examination of the placenta, 15 specimens (6 control cases, 9 PE cases) that were stored as paraffin-embedded sections in Department of Pathology at Osaka Metropolitan University were used for immunohistochemical staining. The placental specimens were collected from the area near the umbilical cord insertion site.

Immunohistochemistry was conducted on 4 µm thick paraffin sections using a two-step indirect method. Following deparaffinization, rehydration was achieved through an autoclave treatment at 121°C for 20 minutes. Subsequently, primary antibody reaction ensued, employing a 100-fold diluted soluble epoxide hydrolase (sEH) antibody (Santa Cruz Biotechnology, Heidelberg, Germany Biotechnology Cat# sc-166961), with overnight incubation at 4°C.

For secondary antibody detection, the Dako REAL EnVision Detection System Peroxidase/DAB+ (cat. no. K5007; Agilent Technologies, Inc., California, America) was utilized, and samples were incubated at room temperature for 3 minutes. Finally, tissue sections were counterstained with hematoxylin at room temperature for 1 minute.

The ImageJ software (ImageJ 180; National Institutes of Health, America) was used to quantify the staining intensity of immunohistochemical staining. The mean optical density of sEH staining in the villous areas of each specimen was calculated, and a comparison of staining intensity was conducted between the control group and the PE group (18).

### 2.6 Statistical analysis

Clinical and metabolomic data were analyzed using the statistical software SPSS (SPSS Inc., an American software company). Metaboanalyst 6.0 was also used to analyze metabolomic data (19).

When the distribution was normal, an unpaired *t*-test was employed. Otherwise, the Mann-Whitney *U* test was used. Differences were considered significant when *p* < 0.05. The interpretation of the correlation coefficients is as follows: 0 < |*r*| < 0.2 indicates no significant correlation, 0.2 ≤ |*r*| < 0.4 indicates a weak correlation, 0.4 ≤ |*r*| < 0.7 indicates a moderate correlation, and |*r*| ≥ 0.7 indicates a strong correlation.

### 2.7 Ethics statement

The use of the samples was approved by the Ethics Committee of the Graduate School of Medicine, Osaka Metropolitan University (Approved Number: 2022-184).

## 3 Result

### 3.1 Study participants

**Table 1** shows an overview of the study participants. The study involved 31 participants, with 20 in the control group and 11 in the PE group. In the PE group, 6 out of 11 cases were diagnosed with FGR. As well, the number of primiparous women was significantly higher in the PE group. However, no significant differences were observed in other perinatal outcomes.

**Table 1:** The table presents maternal characteristics and perinatal outcomes in both groups.

	Controls (n=20)	PE (n=11)	*p* value
	Median (Range) or n (%)	Median (Range) or n (%)	
Maternal characteristics			
Age (years)	34.5 (19 - 43)	32 (26 - 41)	0.338
Primiparous	6 (30)	8 (73)	0.020
ART	3 (15)	2 (18)	0.817
BMI at delivery (kg/m2)	25.67 (19.33 - 33.39)	28.16 (21.83 - 33.78)	0.157
sBP (mmHg)	121.5 (94 - 146)	170 (148 - 188)	<0.001
dBP (mmHg)	74 (54 - 97)	104 (82 - 121)	<0.001
Height (cm)	160 (151.8 - 176.4)	155.0 (146 - 165)	0.244
Perinatal outcomes			
Caesarean section	17 (85)	9 (82)	0.817
Blood loss at delivery (ml)	935 (430 - 1980)	430 (150 - 1110)	0.02
Gestational age (week)	38.4 (36.1 - 41.3)	36.9 (32.0 - 41.0)	0.104
Birth weight (g)	2835 (2334 - 4260)	2345 (1207 - 3165)	0.002
Z-Scores of birth weight (SD)	0.00 (-1.34 - 3.34)	-1.54 (-1.96 - 0.71)	0.003
Umbilical arterial pH	7.264 (7.115 - 7.337)	7.269 (7.196 - 7.284)	0.67
Apgar score at 1 min	8 (5 - 9)	8 (7 - 9)	0.984
Apgar score at 5 min	9 (8-10)	9 (9)	0.823
Male gender	14 (70)	5 (45)	0.179

ART, assisted reproductive technology; BMI, body mass index; saps, systolic blood pressure; dB, diastolic blood pressure; SD, standard deviation.

Continuous variables are represented as median (range).

### 3.2 Comprehensive analysis of plasma samples

**Figure 1:**
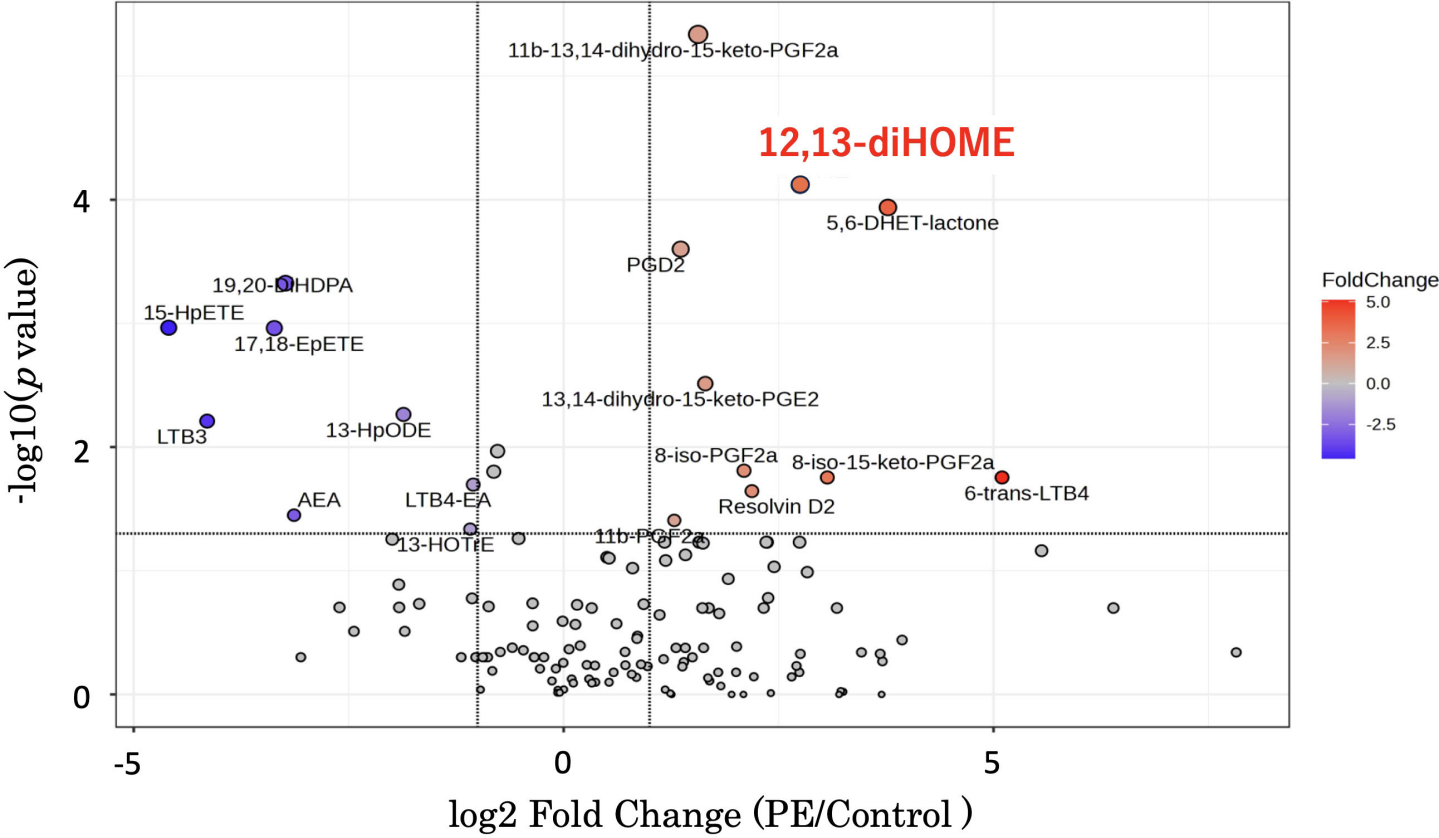
This graph is a volcano plot - a type of scatter plot that is commonly used to display the results of lipid mediator measurements comparing disease and control groups using an LC-MS method package. The x-axis represents the log2 Fold Change, calculated by dividing the mediator levels in the disease group by the control group. A positive log2 Fold Change value indicates an increase in the disease group relative to the control group, while a negative value indicates a decrease. The y-axis shows the negative logarithm (base 10) of the p-value (-log10(p-value)). A higher position on the y-axis reflects a lower p-value, thus indicating a more statistically significant change in the mediator level. Additionally, as you move up the y-axis, the size of the dots representing the data points in the volcano plot increases. 12,13-diHOME is positioned in the upper right quadrant. LC-MS, liquid chromatography-mass spectrometry; 12,13-diHOME, 12,13-dihydroxy-9Z-octadecenoic acid.

Volcano plots (**Figure 1**) were used for supervised data inspection to select the metabolites characterized by the most discriminating power as measured by LC-MS. The x-axis denotes the log2 Fold Change - a logarithmic representation of the ratio of mediator levels in the PE group over the control group. A positive log2 Fold Change indicates an elevation in the PE group, while a negative value suggests a reduction. The y-axis conveys the negative logarithm (base 10) of the *p*-value (-log10(*p*-value)), with higher values indicating stronger statistical significance.

Of particular interest is 12,13-diHOME, which is prominently positioned both high on the y-axis and to the right on the x-axis, thus indicating not only a statistically significant increase in the PE group but also a substantial fold change. The pronounced red coloration of the 12,13-diHOME data point underscores its notable fold increase, reinforcing its potential as a biomarker or a critical player in the pathogenesis of PE (**Figure 1**).

**Figure 2:**
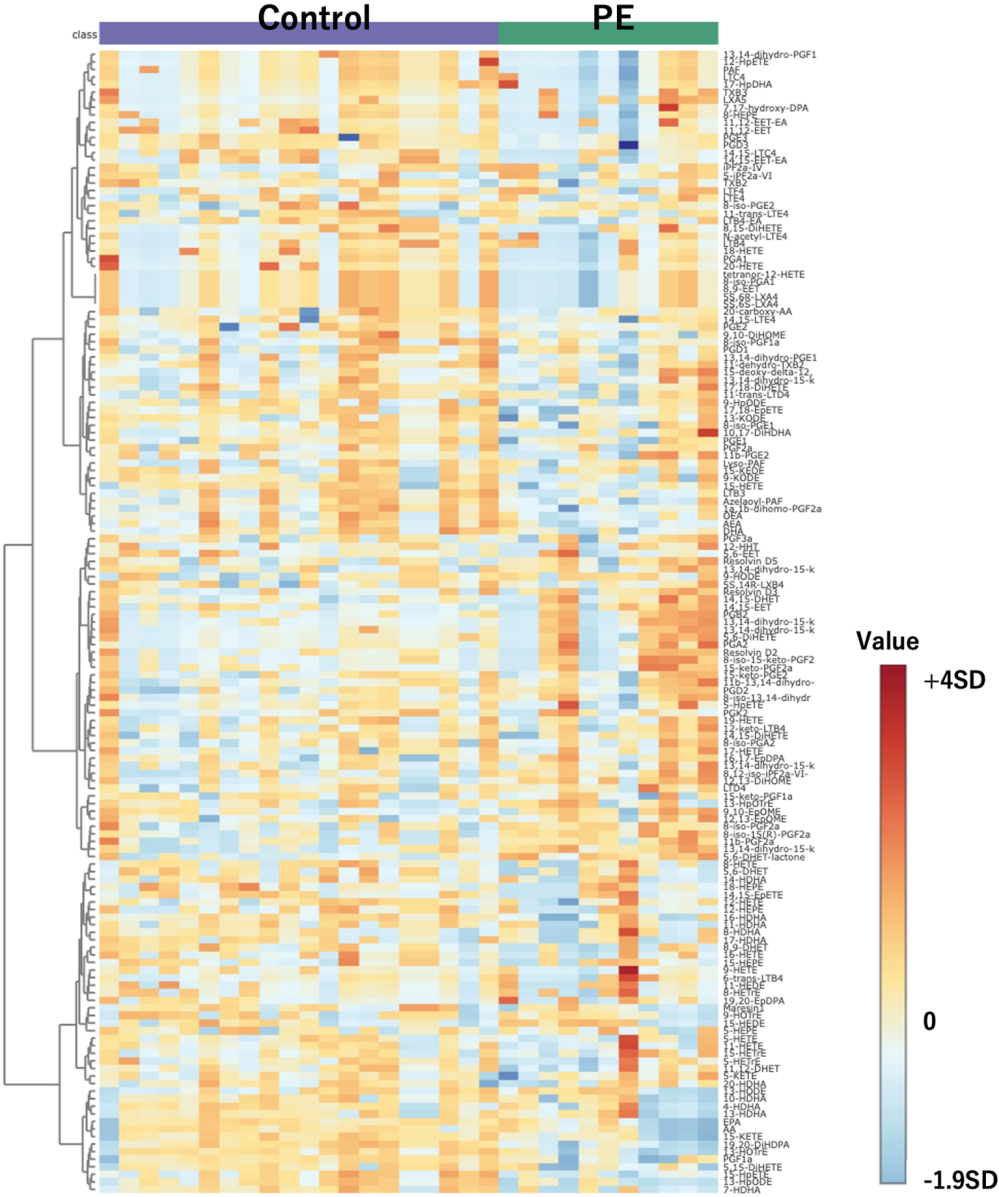
Heatmap of 151 compounds obtained through comprehensive mass spectrometry analysis. Controls are depicted on the left, with PE cases on the right. The color scale bar adjacent to the heatmap represents the standard deviation from the mean. The central color corresponds to a standard deviation of 0, indicating values close to the mean. Warmer colors at the top of the bar indicate values that are above the mean, with increasing intensity representing greater positive standard deviations. Cooler colors at the bottom indicate values below the mean, with greater intensity representing more negative standard deviations. PE, preeclampsia.

**Figure 2** presents a heatmap of the 151 substances detected in this mass spectrometry analysis. The color scale bar adjacent to the heatmap represents the standard deviation from the mean. The central color corresponds to a standard deviation of 0, indicating values close to the mean. Warmer colors at the top of the bar indicate values that are above the mean, with increasing intensity representing greater positive standard deviations. Cooler colors at the bottom indicate values below the mean, with greater intensity representing more negative standard deviations. Rows represent individual compounds that exhibited significant differences in abundance, including 12,13-diHOME among others. These compounds display a range of expression changes, with blue indicating lower and red indicating higher abundance relative to the control group. The color intensity corresponds to the magnitude of the change from the mean of the dataset. Adjacent to the rows, the clustering dendrogram categorizes the compounds based on their expression patterns, thus reflecting the affected metabolic pathways in PE patients. This representation emphasizes the metabolic distinctions that may be related to the pathophysiological processes of PE.

### 3.3 Relationship between maternal blood and umbilical cord blood

**Figure 3:**
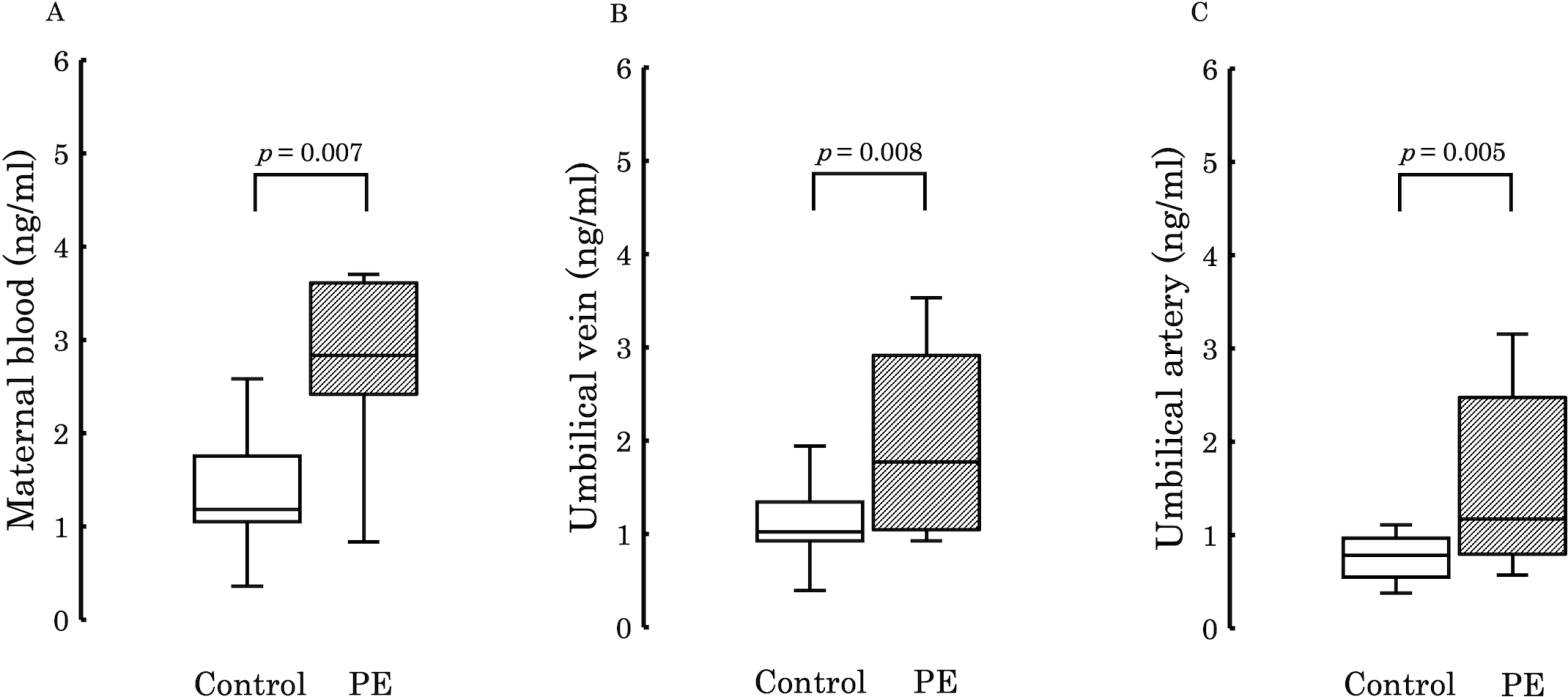
The figure compares the concentrations of 12,13-diHOME quantified in the plasma between the PE and control groups. Significant differences were observed in maternal plasma (**A**; *p* = 0.007), umbilical venous plasma (**B**; *p* = 0.008), and umbilical arterial plasma (**C**; *p* = 0.005), with higher levels detected in the PE group in all cases. PE, preeclampsia; 12,13-diHOME, 12,13-dihydroxy-9Z-octadecenoic acid.

Following comprehensive analysis results revealing significant differences in 12,13-diHOME levels between both groups, we focused on its quantification using 12,13-diHOME-d4 as an internal standard. **Figure 3** illustrates a comparison of the quantification results in maternal plasma, umbilical venous plasma, and umbilical arterial plasma. It demonstrates significantly higher concentrations of 12,13-diHOME in the PE group for each sample type.

**Figure 4:**
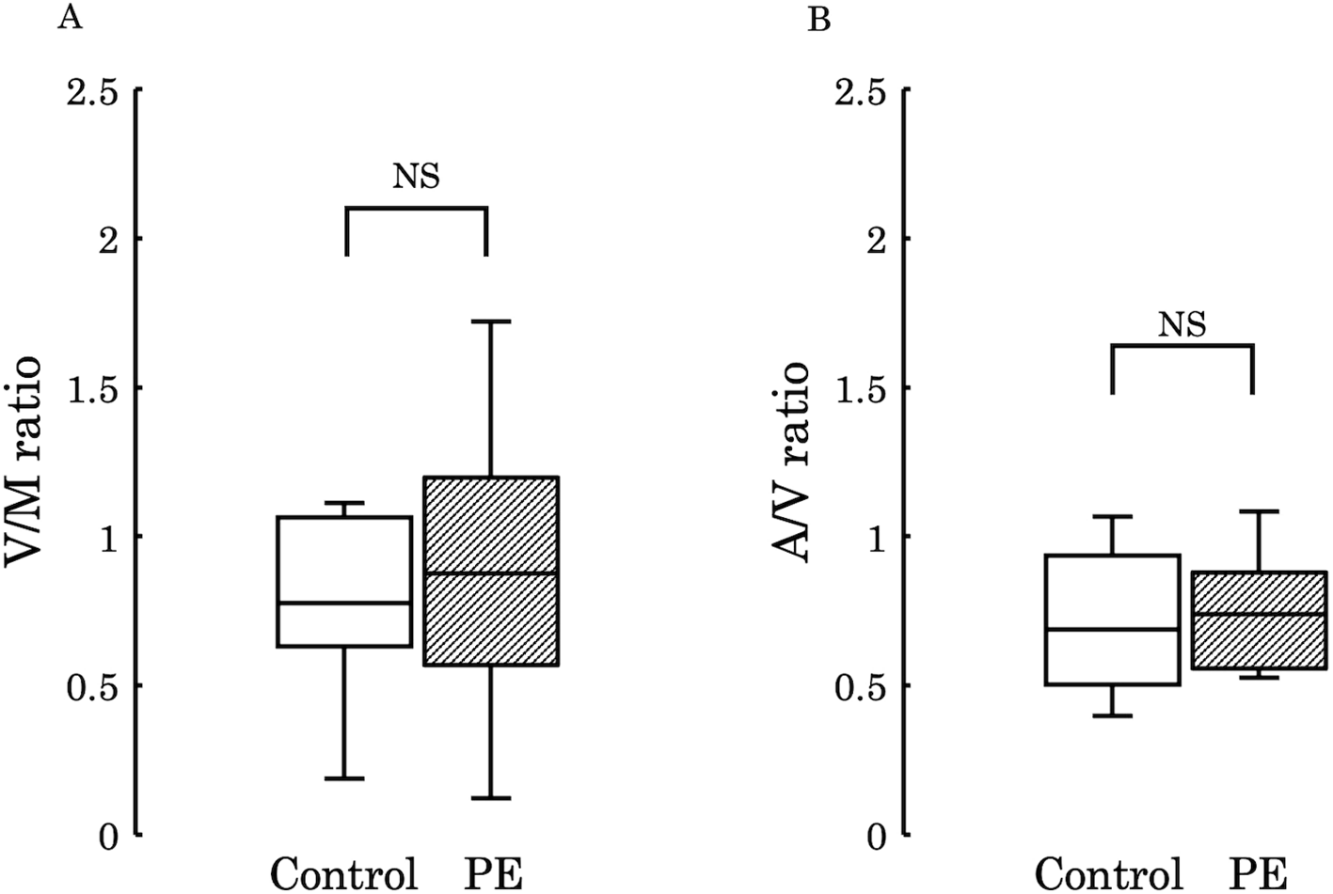
**(A)** The ratio of 12,13-diHOME concentrations in maternal and umbilical venous plasma (V/M ratio) was compared between the control and PE groups, and no significant difference was found (*p* = 0.730). **(B)** The ratio of 12,13-diHOME concentrations between umbilical venous plasma and umbilical arterial plasma (A/V ratio) was compared between the control and PE groups and no significant difference was found (*p* = 0.502). 12,13-diHOME, 12,13-dihydroxy-9Z-octadecenoic acid; NS, not significant.

**Figure 5:**
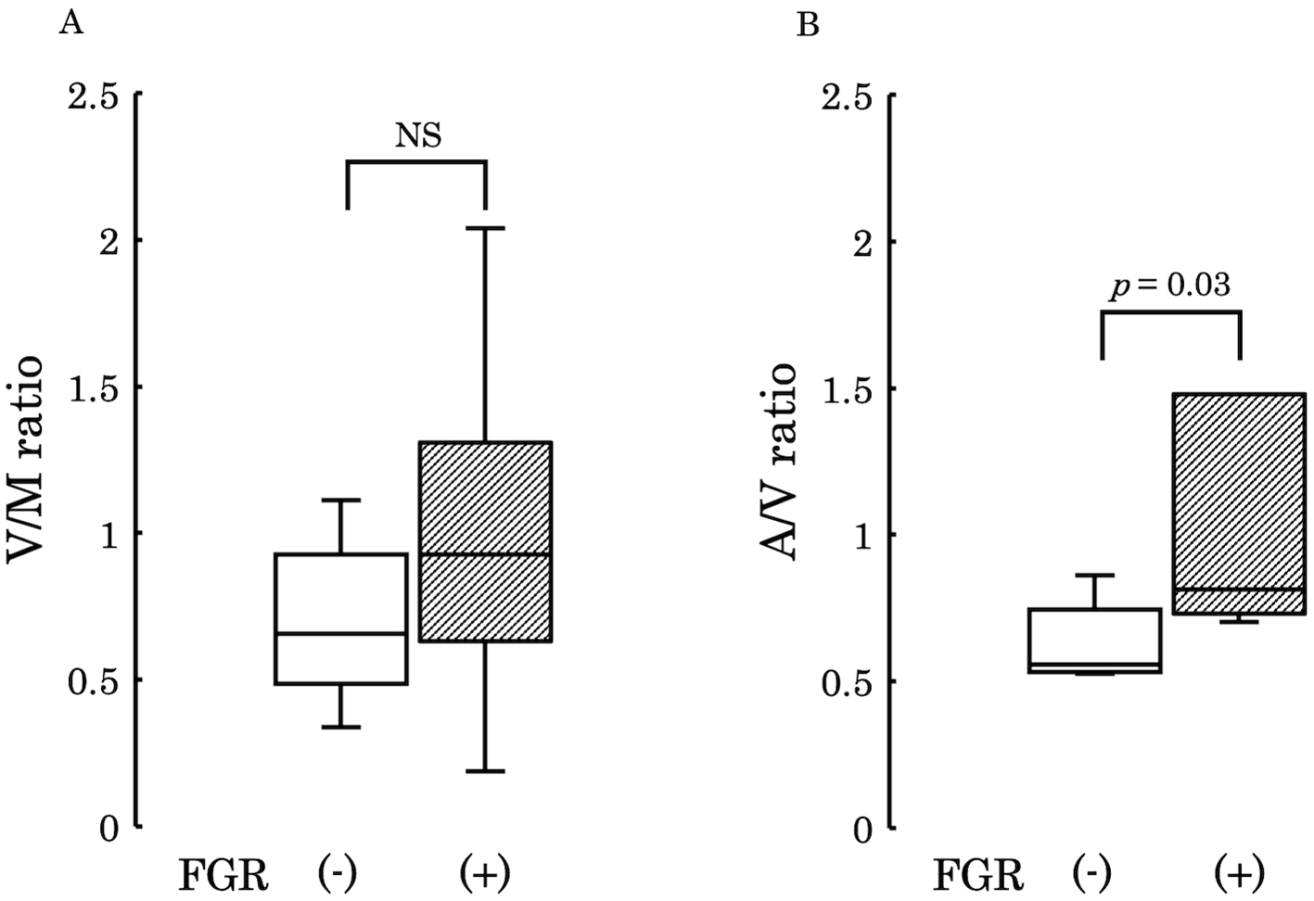
**(A)** The ratio of 12,13-diHOME concentrations in maternal and umbilical venous plasma (V/M ratio) was compared between the PE group with FGR and PE group without FGR and found no significant difference (*p* = 0.329). **(B)** The ratio of 12,13-diHOME concentrations between umbilical venous plasma and umbilical arterial plasma (A/V ratio), thus revealing a significantly higher ratio in the PE group with FGR (*p* = 0.03). PE, preeclampsia; FGR, fetal growth restriction; 12,13-diHOME, 12,13-dihydroxy-9Z-octadecenoic acid; NS, Not Significant.

Subsequently, to examine the variations in 12,13-diHOME concentration from the maternal to the umbilical vein and from the umbilical vein to the umbilical artery, we conducted comparisons between the umbilical venous plasma to the maternal plasma ratio (V/M ratio) and the umbilical arterial plasma to the umbilical venous plasma ratio (A/V ratio) across the two groups. No significant differences were observed in these ratios between the groups (V/M ratio: *p* = 0.73, A/V ratio: *p* = 0.50) (**Figure 4**). However, when comparing PE with FGR to PE without FGR, no significant difference was found in the V/M ratio (*p* = 0.329) (**Figure 5A**), whereas the A/V ratio was significantly higher in the PE with FGR group compared to the PE without FGR group (*p* = 0.030) (**Figure 5B**).

### 3.4 Correlation between 12,13-diHOME concentrations and birth weight, gestational age, and BMI

**Figure 6:**
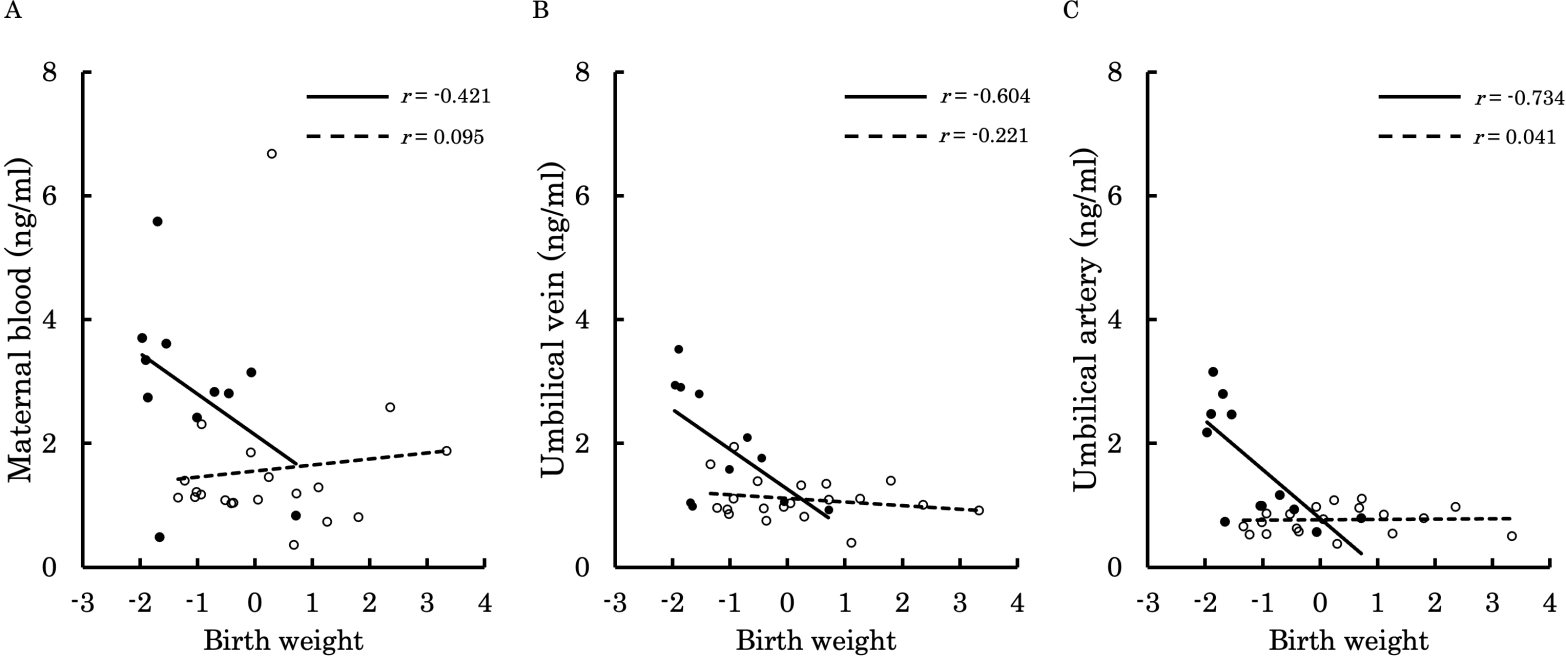
This figure shows the relationship between the concentration of 12,13-diHOME (ng/ml) in maternal plasma, umbilical venous plasma, and umbilical arterial plasma and birth weight Z-Scores, represented with a scatter plot and a regression curve. The figure also includes Pearson’s correlation coefficient. Filled circles and thick lines represent the concentrations and values of each parameter in the PE group, along with the corresponding regression line. Empty circles and dashed lines represent the concentrations and values of each parameter in the control group, including the corresponding regression line. Each parameter is presented: maternal plasma **(A)**, umbilical venous plasma **(B)**, and umbilical arterial plasma **(C)**. 12,13-diHOME, 12,13-dihydroxy-9Z-octadecenoic acid; PE, preeclampsia.

**Figure 7:**
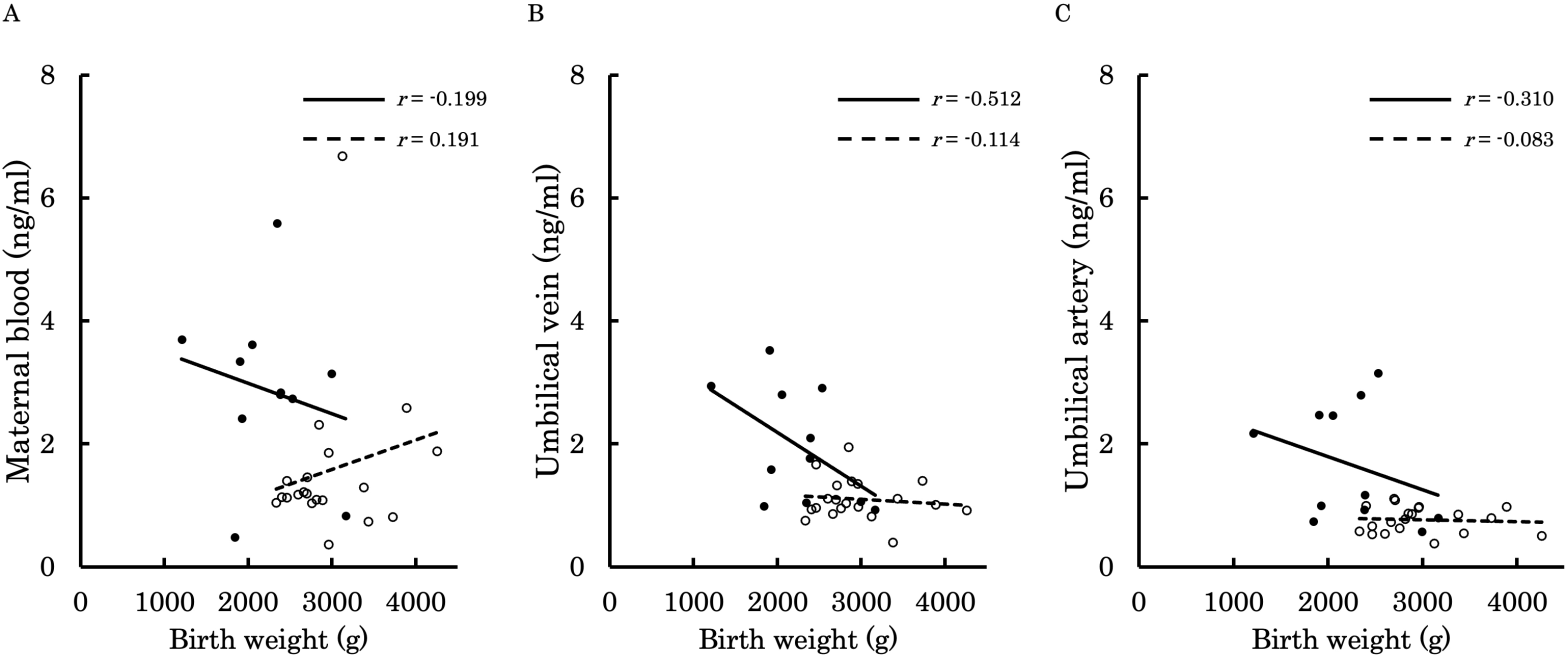
This figure shows the relationship between the concentration of 12,13-diHOME (ng/ml) in maternal plasma, umbilical venous plasma, and umbilical arterial plasma and birth weight **(g)**, represented with a scatter plot and a regression curve. The figure also includes Pearson’s correlation coefficient. Filled circles and thick lines represent the concentrations and values of each parameter in the PE group, along with the corresponding regression line. Empty circles and dashed lines represent the concentrations and values of each parameter in the control group, including the corresponding regression line. Each parameter is presented: maternal plasma **(A)**, umbilical venous plasma **(B)**, and umbilical arterial plasma **(C)**. 12,13-diHOME, 12,13-dihydroxy-9Z-octadecenoic acid; PE, preeclampsia.

**Figure 8:**
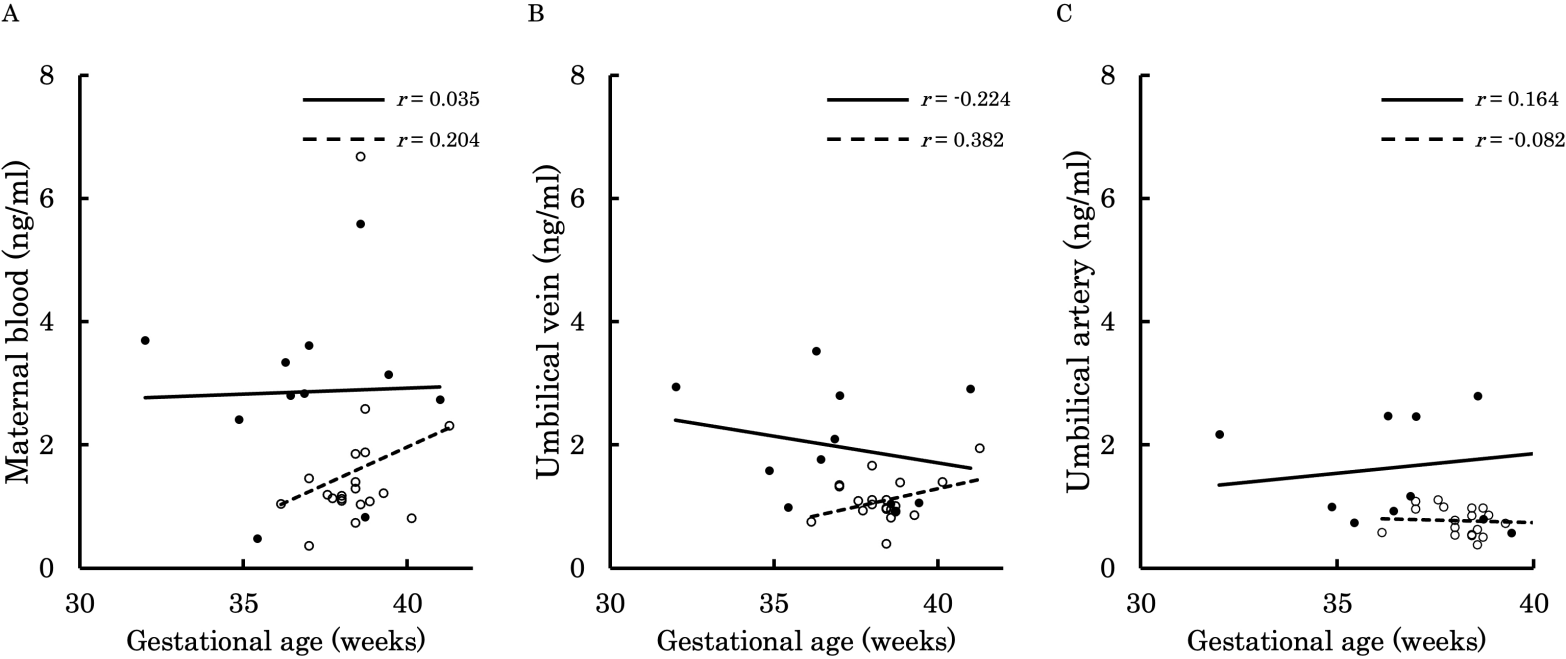
This figure shows the relationship between the concentration of 12,13-diHOME (ng/ml) in maternal plasma, umbilical venous plasma, and umbilical arterial plasma and gestational age (weeks), represented with a scatter plot and a regression curve. The figure also includes Pearson’s correlation coefficient. Filled circles and thick lines represent the concentrations and values of each parameter in the PE group, along with the corresponding regression line. Empty circles and dashed lines represent the concentrations and values of each parameter in the control group, including the corresponding regression line. Each parameter is presented: maternal plasma **(A)**, umbilical venous plasma **(B)**, and umbilical arterial plasma **(C)**. 12,13-diHOME, 12,13-dihydroxy-9Z-octadecenoic acid; PE, preeclampsia.

**Figure 9:**
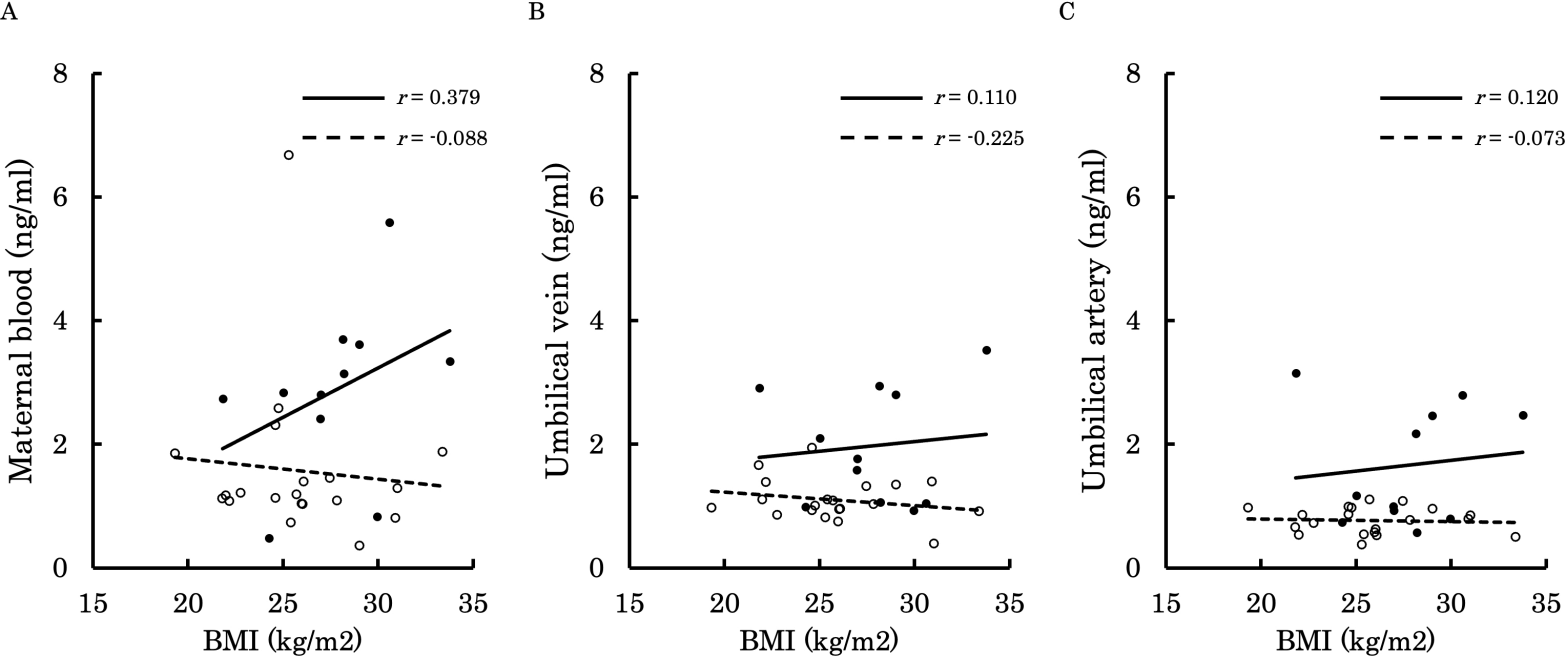
This figure shows the relationship between the concentration of 12,13-diHOME (ng/ml) in maternal plasma, umbilical venous plasma, and umbilical arterial plasma and BMI at delivery (kg/m²), represented with a scatter plot and a regression curve. The figure also includes Pearson’s correlation coefficient. Filled circles and thick lines represent the concentrations and values of each parameter in the PE group, along with the corresponding regression line. Empty circles and dashed lines represent the concentrations and values of each parameter in the control group, including the corresponding regression line. Each parameter is presented: maternal plasma **(A)**, umbilical venous plasma **(B)**, and umbilical arterial plasma **(C)**. 12,13-diHOME, 12,13-dihydroxy-9Z-octadecenoic acid; PE, preeclampsia; BMI, body mass index.

**Figures 6**–**9** shows the correlations between the concentrations of 12,13-diHOME in maternal plasma, umbilical venous plasma and umbilical arterial plasma, and various parameters (birth weight, gestational age and BMI). Regarding the correlation between 12,13-diHOME and birth weight Z-Scores in the control group, there were weak or no correlations. However, in the PE group, moderate negative correlations were observed in maternal plasma (*r* = -0.421) and umbilical venous plasma (*r* = -0.604), while a strong negative correlation was observed in umbilical arterial plasma (*r* = -0.734). Regarding the correlation with birth weight (g) in the PE group, a moderate negative correlation was found in umbilical venous plasma (*r* = -0.512). No correlations were observed in the control group. Regarding the correlation between gestational age at delivery and 12,13-diHOME, a positive correlation was found in maternal plasma of the control group (*r* = 0.204). Regarding the correlation between BMI at delivery and 12,13-diHOME, a positive correlation was found only in maternal plasma of the PE group (*r* = 0.379).

### 3.5 Immunohistochemistry of placenta

**Figure 10:**
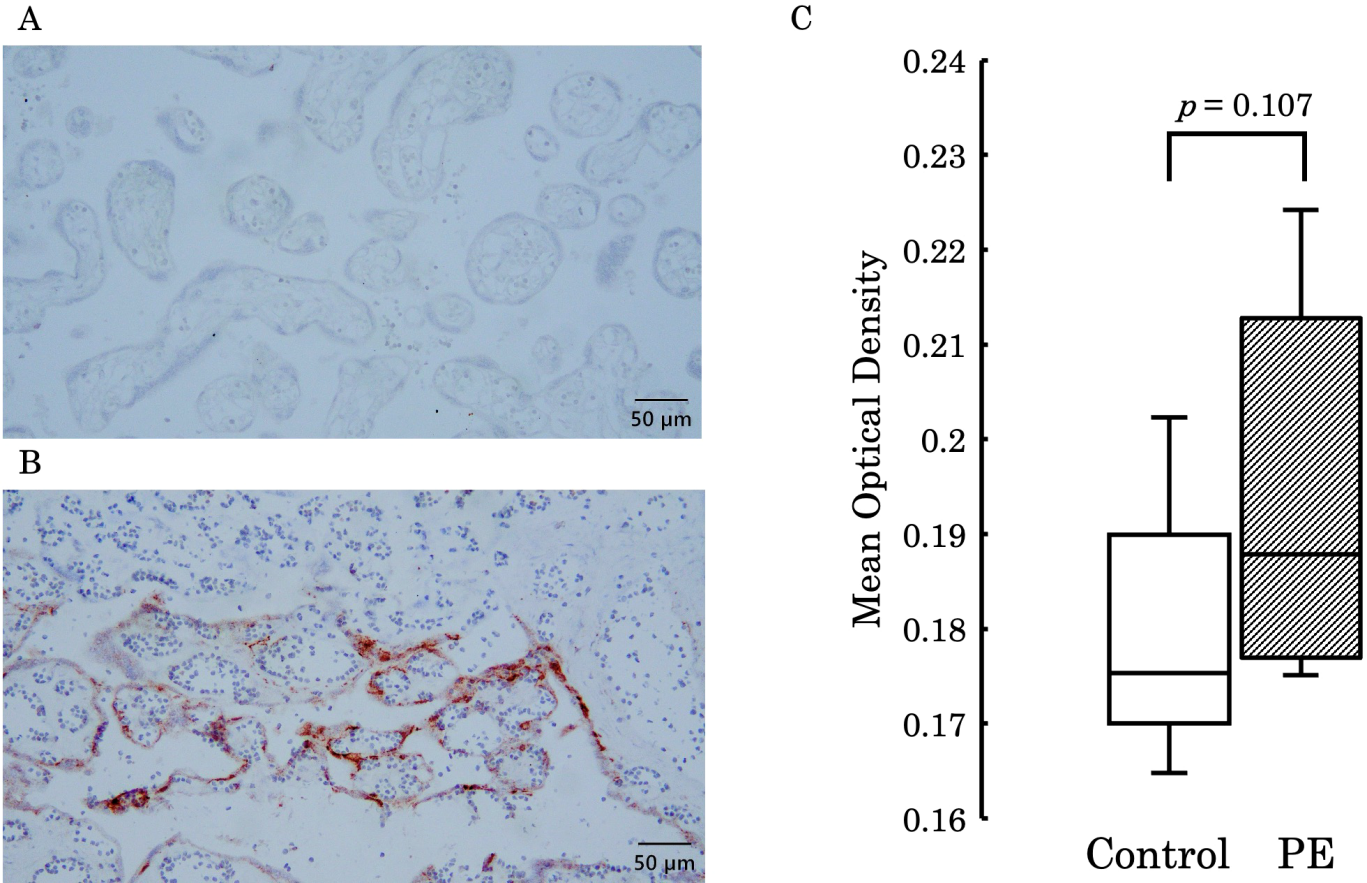
**(A)** Represents the negative control, and **(B)** shows staining in the outer layers of the placental villi. **(C)** Presents a comparison of the mean optical density between the control group and the PE group, with no significant difference observed between the two groups (*p* = 0.107). PE, preeclampsia.

Immunohistochemical staining was performed to compare the differences in soluble epoxide hydrolase sEH expression in the placental villi. **Figure 10** shows the expression levels of sEH in the placental villi. (A) represents the negative control, and (B) shows one of the stained placental samples. The stained areas were distributed in the outer layers of the villi regardless of intensity. Furthermore, (C) presents a comparison of the mean optical density between the control group and the PE group. Although there was no significant difference in staining intensity between the two groups (*p* = 0.107), a stronger tendency was observed in the PE group.

## 4 Discussion

In fetal circulation, oxygen- and nutrient-rich blood from the placenta is transported to the fetus through the umbilical vein (20). This blood travels from the fetal heart through the aorta, delivering oxygen and nutrients to the entire body (20). The blood then returns to the placenta via the umbilical arteries. In the placenta, carbon dioxide and other waste products are exchanged and enter the maternal circulation (20). Our study firstly revealed the characteristic changes in the lipid profile of PE patients using comprehensive analysis with LC-MS (**Figure 1**). As for the plasma levels of 12,13-diHOME, striking differences between a normal pregnancy and PE were also demonstrated in both maternal and umbilical cord blood. Furthermore, PE with FGR patients showed a significant increase in the plasma 12,13-diHOME level in the umbilical arterial/venous ratio (**Figure 5**). Of note, there were significant negative correlations between the Z-Scores of birth weight and the plasma levels (**Figure 6**).

Various lipid metabolites are generated from linoleic acid (an omega-6 fatty acid) through catalytic reactions by cytochrome P450 (21). Some of these metabolites, epoxides, are further metabolized by soluble epoxide hydrolase (sEH) to form diHOMEs (21). These lipid metabolites are known as lipokines, and they play pivotal roles in signal transmission (22). 12,13-diHOME, which is released from brown adipose tissue (BAT), is shown to function as a signaling mediator, thus augmenting metabolism after exercise or cold exposure with the enhancement of the thermogenic activity in BAT (22, 23). Furthermore, in the process of nitric oxide (NO) production, 12,13-diHOME serves as a promotor of the phosphorylation of protein kinase B, which in turn enhances the phosphorylation of endothelial NO synthase (eNOS) (11). These accumulating data of 12,13-diHOME bioactivity suggest its involvement in the adaptation and modification of pregnancy-related phenomena; however, there are few reports so far as to the association of 12,13-diHOME in perinatal outcomes.

The pathogenesis of PE is thought to be caused by placental ischemia (24). Shallow cytotrophoblast invasion into the uterine spiral artery causes the defect of arterial remodeling, leading to impaired placental perfusion (25). As a consequence of placental ischemia and structural damage, the placenta releases various factors into the maternal circulation and triggers oxidative stress to the syncytiotrophoblast, thus resulting in the production of anti-angiogenetic factors and proinflammatory cytokines (26). These deleterious circumstances further lead to the formation of atherosis in spiral arteries, equivalent to the atherosclerotic lesion found in the coronary arteries (26–28). At the onset of acute myocardial infarction in patients with type 2 diabetes mellitus, an elevated plasma level of 12,13-diHOME was observed by Cao et al., indicating a possible role as a marker of metabolic alteration (29). Considering the extent to which 12,13-diHOME was elevated in the PE group in our study, compared to the control group, it is possible that the elevated 12,13-diHOME observed in the PE group reflects maternal vascular damage due to placental ischemia. Furthermore, Levan et al. demonstrated that elevated 12,13-diHOME concentration may trigger inflammation and impede immune tolerance by decreasing regulatory T cells in animal models (30). In fact, they also found the association between an increased copy number of bacterial epoxide hydrolase and 12,13-diHOME in the feces of neonates, who were associated with a high probability of developing atopy, eczema, or asthma during childhood (30). Considering this together with the immunological involvement of regulatory T cells in the pathogenesis of PE (31, 32), the elevated maternal concentration of 12,13-diHOME may be complicit in damaging the maintenance of an immunologically normal pregnancy by inhibiting the regulatory T cell function, thereby causing PE. A study comparing the blood concentration of 12,13-diHOME between lowland and highland areas examined the changes in 12,13-diHOME levels under tissue hypoxia. It reported that sEH upregulated in hypoxic conditions, thus leading to an increase in blood levels of 12,13-diHOME. The pathophysiology of PE involves placental hypoxia, and the elevated levels of 12,13-diHOME in maternal and umbilical cord blood due to PE may reflect this pathological condition (33). 12,13-epoxyoctadecaenoic acid (12,13-EpOME) is hydrolyzed by sEH into 12,13-diHOME (21). The expression of sEH increases under oxidative stress, inflammation (34), and hypoxic conditions (33). In our study, there was no significant difference in 12,13-EpOME concentrations between the two groups. It is well-known that PE is caused by placental ischemia (24). The resulting oxidative stress and hypoxia likely lead to an increase in sEH expression, enhancing the conversion of 12,13-EpOME to 12,13-diHOME, which resulted in a significantly higher concentration of 12,13-diHOME in the PE group. Although there was no significant difference in sEH expression levels in the placenta between the groups, the PE group showed a trend toward higher expression of sEH. Therefore, further investigation with an increased number of cases is needed to explore this trend in more detail.

FGR can be caused by a variety of factors, including infection, chromosomal abnormalities, genetic abnormalities, race, and placental insufficiency (35–38). The most common cause is placental insufficiency, which is thought to account for 70% of all FGRs (39). As far as we know, there seems to be one study of 12,13-diHOME levels in umbilical cord blood and an association with birth weight (40). Umeda et al. showed a negative correlation of 12,13-diHOME concentration with birth weight but did not take into account the weeks of gestation, nor did they consider the cause of low birth weight (40). Therefore, we limited our investigation to FGR due to placental insufficiency, which can be clearly defined by antenatal ultrasound scanning.

The present study showed that the median ratio of the umbilical artery to the umbilical vein in control tended as <1 (**Figure 4**), thus suggesting that the fetus consumes and utilizes 12,13-diHOME derived from maternal circulation in a normal physiological status. This concept may be plausible considering that fetal movement itself requires energy. Interestingly, the umbilical arterial/venous ratio of 12,13-diHOME was significantly elevated in FGR of PE compared to that of normally grown fetuses in PE. This finding suggests that severe FGR patients will suffer from more deleterious intra-uterine circumstance due to placental ischemia, and this speculation may be supported the results that the Z-Scores of birth weight showed a negative correlation with 12,13-diHOME concentration (**Figure 5**). A study investigating the concentration of 12,13-diHOME in breast milk and infant development reported that the concentration of 12,13-DiHOME in breast milk showed a positive correlation with birth weight, but subcutaneous fat and overall body fat decreased after one month. Furthermore, the increase in Weight-for-Length and BMI Z-scores from 0 to 6 months was lower, thus suggesting an association with the postnatal suppression of obesity (41). In our study, the reason for the negative correlation between maternal blood and the umbilical cord blood concentrations of 12,13-diHOME and birth weight may be due to the promotion of fat uptake by 12,13-diHOME, therefore leading to weight suppression. However, to clarify these mechanisms, future research using animal models during pregnancy, as well as studies utilizing samples from children not only from umbilical cord blood but also a few months after birth, will be necessary.

There are some limitations in our study. Firstly, we investigated a relatively small number of healthy pregnant women and patients with PE. Secondly, 12,13-diHOME is a metabolite of the omega-6 fatty acid linoleic acid, which is not synthesized endogenously in the body and can be obtained through oral intake. Therefore, we cannot negate the potential influence of dietary habits, which we did not investigated in this study. Furthermore, in this study, linoleic acid was not included in the method package used for measurement, so it could not be measured directly. Thirdly, the physiological variation in 12,13-diHOME concentrations throughout pregnancy, as well as in primiparous and multiparous women, was not evaluated.

In conclusion, our study firstly demonstrated the characteristics of the maternal and umbilical cord blood concentrations of 12,13-diHOME in healthy women and those with PE, thus suggesting that and elevated 12,13-diHOME may reflect the progression of placental ischemia due to the pathogenesis of PE. Our findings add a new insight into perinatal lipidomics - that 12,13-diHOME may serve as a marker for the severity of preeclampsia.

## Data Availability

The raw data supporting the conclusions of this article will be made available by the authors, without undue reservation.
